# Controlled Release of Dutasteride from Biodegradable Microspheres: *In Vitro* and *In Vivo* Studies

**DOI:** 10.1371/journal.pone.0114835

**Published:** 2014-12-26

**Authors:** Xiangyang Xie, Yanfang Yang, Qiang Chi, ZhiPing Li, Hui Zhang, Ying Li, Yang Yang

**Affiliations:** 1 Department of Pharmacy, Wuhan General Hospital of Guangzhou Military Command, Wuhan, PR China; 2 Beijing Key Laboratory of Drug Delivery Technology and Novel Formulation, Institute of Materia Medica, Chinese Academy of Medical Sciences & Peking Union Medical college, Beijing, PR China; 3 Department of Pharmacy, The 215th Clinic of 406th Hospital of the Chinese People's Liberation Army, Dalian, PR China; 4 Beijing Institute of Pharmacology and Toxicology, Beijing, PR China; The University of Tennessee Health Science Center, United States of America

## Abstract

The aim of the present work was to study the *in vitro/in vivo* characteristics of dutasteride loaded biodegradable microspheres designed for sustained release of dutasteride over four weeks. An O/W emulsion-solvent evaporation method was used to incorporate dutasteride, which is of interest in the treatment of benign prostatic hyperplasia (BPH), into poly(lactide-co-glycolide) (PLGA). A response surface method (RSM) with central composite design (CCD) was employed to optimize the formulation variables. A prolonged *in vitro* drug release profile was observed, with a complete release of the entrapped drug within 28 days. The pharmacokinetics study showed sustained plasma drug concentration-time profile of dutasteride loaded microspheres after subcutaneous injection into rats. The *in vitro* drug release in rats correlated well with the *in vivo* pharmacokinetics profile. The pharmacodynamics evaluated by determination of the BPH inhibition in the rat models also showed a prolonged pharmacological response. These results suggest the potential use of dutasteride loaded biodegradable microspheres for the management of BPH over long periods.

## Introduction

Benign prostatic hyperplasia (BPH) is age related proliferative diseases, which is extremely common medical condition in aged men 50 years or older [1], [2]. For many aged men, the prostatic enlargement and bladder outlet obstruction, could lead to severe lower urinary tract symptoms, such as decreased maximum urinary flow rate, urinary frequency and urgency [3]. Patients with BPH not only have their life quality affected, but also at greater risk for acute urinary retention and BPH-related surgery [4]. Thus, the management of BPH remains a major challenge in andrology.

Currently, dutasteride is widely used in the clinical to treat BPH. According to the report of Roehrborn et al., patients with long-term (4-year) treatment of dutasteride were proved to have continuing improvements in symptoms and peak urinary flow [5]. At present, dutasteride is available in the market as soft gelatin capsule (0.5 mg), and daily oral administration is required. Though oral administration is convenient for most patients, it may be difficult for some elderly patients who are suffering from hypomnesis or mental disorder to secure the therapy. A sustained release dosage form for long periods of time avoids daily administration, and is therefore the best way to improve patient compliance and secure the therapeutic efficiency.

Biodegradable injectable microspheres have been studied widely in the last 30 years. They can prolong the duration of a drug effect significantly, reduce the frequency of administration and hence improve patient compliance. The total dose and some adverse reactions may be reduced because of a steady plasma concentration [6]. Moreover, there is also no need for them to be implanted by surgical operation and removed after the drug is completely released. Among the various biodegradable polymers, poly (lactic-co-glycolic acid) (PLGA) has been used in several FDA approved parenteral microspheres that are currently on the market (e.g. Lupron Depot, Trelstar, Zoladex, Vivitrol and Risperdal Consta). Due to the safety profile and properties in facilitating controlled drug release of PLGA, we have been interested in developing a PLGA microsphere system that can be used for continuous delivery of dutasteride for BPH patients.

In this paper, a newly developed controlled release system based on PLGA microspheres loaded with dutasteride was prepared and evaluated. The microspheres were prepared by O/W emulsification solvent evaporation method. Response surface methodology (RSM) was utilized to optimize the formulation with three response variables. Besides the evaluation of physicochemical characteristics, the *in vitro* release of the PLGA microspheres was also investigated. An analytical method based on LC-MS/MS technique was developed for the simultaneous determination of dutasteride in blood samples. The correlation between *in vitro* and *in vivo* release was established. Finally, the BPH inhibition effects of microspheres on rat models were investigated.

## Materials and Animals

### Ethics Statement

This study did not involve non-human primates. All experiments described in this study were performed in full accordance with the guidelines for animal experiments released by the National Institute of Animal Health. This study is approved by the Animal Care and Use Ethics Committee of the 215th Hospital (ETHICS CODE Permit NO.SCXK (Liao) 2014-0023).

### Materials

PLGA (Mw 15000 Da, lactide/glycolide ratio, 75/25) were supplied by the Institute of Chemistry (Wuhan University, China) with capped end groups as ester. Dutasteride (> 99% purity) was purchased from Wuhan Xianbao Bio-pharmaceutical Company (Hubei, China). Testoviron injections were purchased from Shanghai General Pharmaceutical Company (Shanghai, China). RIA kits for measurement of testosterone and dihydrotestosterone (DHT) levels were purchased from the institute of Jiancheng Biological Engineering (Nanjing, China). Polyvinyl alcohol (PVA-124) and dichloromethane (DCM) were obtained from Beijing Chemical Reagents Company. All other materials or solvents were of reagent or analytical grade.

### Experiment and methods

#### Preparation of microspheres

An O/W emulsion solvent evaporation method was used to prepare dutasteride PLGA microspheres. An amount of PLGA (99.43, 142.27, 199.50, 259.00 and 299.57 mg) and dutasteride (theoretical drug content: 1.99, 3.21, 5.00, 6.80 and 8.02%, w/v) were added to 1 mL of DCM. After being completely dissolved, the mixture was dropped slowly into 5 mL of PVA-124 aqueous solution (concentration: 3.04, 3.63, 4.50, 5.37 and 5.96%, w/v) and then the mixture was emulsified by using a propeller stirrer (SXJQ-1, Zhengzhou, China) at 1000 rpm for 10 min at 25°C. The mixture emulsion was poured into 200 mL deionized water, stirring at 450 rpm for 6 h to evaporate the organic solvent. The hardened microspheres were collected by centrifuging at 8,000 rpm for 5 min, and washed three times with 10 mL deionized water, finally dried under vacuum at room temperature to obtain free flowing powder.

The batch used in the animal study was prepared applying larger amounts of reagents, even though their relative composition was kept identical.

#### Formulation optimization

RSM as a tool of designing experiments is widely utilized in the development and optimization of drug delivery systems [7]. Central composite design (CCD) enables several independent variables to be investigated at the same time using a relatively small number of experiments [8], [9]. Hence, a central composite design-response surface methodology (CCD-RSM) was used to systemically investigate the effect of independent/dependent variables and optimize the formulation.

In a preliminary study, the key factors that would influence the quality of the microspheres were investigated by a single factor exploration. As shown in Table 1, as for each three rows (group), the values of a factor were various (the number letters are bold), and the values of the left factors were fixed. One-way analyses of variance (ANOVA) were performed to evaluate the difference among the three rows (in a group). As displayed in the Table 1, these factors such as stirring speed, emulsified time and Oil/Water phase ratio demonstrated no statistical influence on the quality indexes of the microspheres. Therefore, the left factors, such as PVA concentration (X_1_, w/v), PLGA content in the oil phase of the emulsion (X_2_, w/v), and theoretical drug content (X_3_, w/w) were chosen as the independent variables (factors) in the preparation of microspheres. Drug encapsulation efficiency (Y_1_), mean diameter (Y_2_) and cumulative drug release F_1d_ after 1 day (Y_3_) were selected as dependent variables. In the preparation process, all the remaining factors, such as stirring speed, emulsified time and Oil/Water phase ratio were kept constant.

**Table 1:** Formulation investigation for preliminary study (n = 3).

No.	PVA concentration (%, w/v)	Theoretical drug content (%,w/w)	Stirring speed (rpm)	PLGA concentration (mg·mL^−1^)	Emulsified time (min)	Oil: Water (v/v)	Appearance	Encapsulation efficiency (%)	F_1d_ (%)	F_28d_ (%)
1	**3.0**	5.0	1000	100	10	1∶10	Spherical	78.6±3.2	8.03±4.1	83.1±4.7
2	**4.5**	5.0	1000	100	10	1∶10	Spherical	83.3±1.2*	10.1±6.8	87.5±2.2
3	**6.0**	5.0	1000	100	10	1∶10	Spherical	70.4±2.1	15.7±7.2	88.1±3.9
4	4.5	**2.0**	1000	100	10	1∶10	Spherical	90.6±3.6*	7.6±4.4	81.4±4.1
5	4.5	**5.0**	1000	100	10	1∶10	Spherical	83.3±1.2*	10.1±6.8	87.5±2.2
6	4.5	**8.0**	1000	100	10	1∶10	Spherical	65.1±4.3*	17.9±7.2	85.2±3.1
7	4.5	5.0	**800**	100	10	1∶10	Spherical	85.8±2.0	11.6±5.6	84.7±2.8
8	4.5	5.0	**1000**	100	10	1∶10	Spherical	83.3±1.2	10.1±6.8	87.5±2.2
9	4.5	5.0	**1200**	100	10	1∶10	Spherical	81.0±2.3	13.6±7.1	79.0±1.9
10	4.5	5.0	1000	**100**	10	1∶10	Spherical	83.3±1.2	10.1±6.8	87.5±2.2
11	4.5	5.0	1000	**200**	10	1∶10	Spherical	86.4±1.0	9.5±4.3	79.7±1.6
12	4.5	5.0	1000	**300**	10	1∶10	Spherical	93.3±2.4*	8.4±5.8	82.9±3.3
13	4.5	5.0	1000	100	**10**	1∶10	Spherical	83.3±1.2	10.1±6.8	87.5±2.2
14	4.5	5.0	1000	100	**20**	1∶10	Spherical	82.1±1.7	11.3±6.4	88.1±2.8
15	4.5	5.0	1000	100	**40**	1∶10	Spherical	81.5±1.9	10.2±7.1	86.2±2.4
16	4.5	5.0	1000	100	10	**1∶10**	Spherical	83.3±1.2	10.1±6.8	87.5±2.2
17	4.5	5.0	1000	100	10	**1∶15**	Spherical	82.6±2.1	11.1±7.2	88.1±3.1
18	4.5	5.0	1000	100	10	**1∶20**	Spherical	82.4±1.8	10.5±6.3	87.2±2.7

F_1d_: Cumulative *in vitro* drug release after 1 day; F_28d_: Cumulative *in vitro* drug release after 28 days.

*Significant different (p<0.05) when compared with others in a group (factor).

Experimental design was formulated according to CCD tool of RSM using Design-Expert 7.0 software (Stat-Easy In., MN, USA). Open the software, click on the “Response Surface” folder tab to show the designs available for RSM, and select the “Central Composite” design. Click on the down arrow in the “Numeric Factors” entry box and Select 3. Click the “Options” at the bottom of the screen, choose the “Rotatable” design with the axial (star) points set at 1.68719 coded units from the center - a conventional choice for the CCD. For “Center points” enter the number 6 - the normal default value and press the “Tab” key. Type in the details for factor Name (A, B, C), Units and levels for low (−1) and high (+1) (as shown in Table 2), then the values of (±) alpha (1.687) will be produced automatically by the software. There are five levels for a factor generated by the software automatically. The following steps were operated according to the tutorial of the software.

**Table 2:** Levels of formulation parameters used in experiment.

	Levels				
Factor	−1.687	−1	0	+1	+1.687
X_1_ (%)	3.04	3.63	4.50	5.37	5.96
X_2_ (mg·mL^−1^)	99.43	140.00	199.50	259.00	299.57
X_3_ (%)	1.99	3.21	5.00	6.80	8.02

There is a “decimal accuracy' constrains issue should be notified. Taken the “theoretical drug content” for example, when input the two decimal retained (accuracy usually used in pharmaceutics research) numerical values (3.21, 6.80) into the software, it will display the numbers of 1.99 (level −1.687) and 8.02 (level +1.687). Although the ideal values of −1.687 and +1.687 level were 2.00 and 8.00, we could not obtain such values in the software due to constrains in decimal accuracy, so we chosen the approximate ones (1.99 and 8.02).

#### Determination of dutasteride content

25 mg of dutasteride-loaded microspheres were dissolved in 2 mL of an acetonitrile-water solution (9∶1, v/v), diluted to 20 mL with water, and vortexed for 1 min. The resulting solution was then left to stand undisturbed for 5 min. The resulting solution was centrifuged at 10,000 rpm for 10 min. The concentration of dutasteride in the supernatant was determined by an HPLC system (Waters 2487, Waters, USA), which consisted of a pump and a UV-Vis detector set at 220 nm. The stationary phase was composed of a reversed phase C_18_ column (5 µm, 4.6 mm×250 mm, Agela technologies, China), and the analysis temperature was 25°C. The mobile phase consisted of methanol and water at a ratio of 80∶20 (v/v); the flow rate was 1 mL/min, and the injection volume was 20 µL.

The polymers did not interfere with absorbance of the drug at the specified wavelength. The drug loading (DL) and encapsulation efficiency (EE) of the microspheres were calculated as follows: 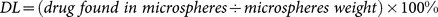


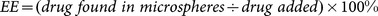



All of the measurements were conducted in triplicate.

#### Particle size analysis

A light-scattering particle size analyzer (Ls800, OMEC, China) was used to determine the size distribution of the prepared microspheres. To do this, microspheres were suspended in 0.5% (w/v) sodium carboxymethyl cellulose solution [10]. Particle size was measured in triplicate.

#### Injectability

Approximately 10 mg PLGA microspheres were suspended in 1 mL dispersed solution (containing 0.5% CMC-Na and 0.05% Tween-80). Two milliliter syringes, fitted with needles of different inner diameter, containing the microsphere suspensions were placed in an Instron 4501 instrument (Instron, MA, USA) to measure ejection force [11].

#### Microscopic observations

For the shape and surface analysis, the microspheres were mounted onto an aluminum stub using double-sided adhesive tape, and then sputter coated with a layer of gold under vacuum before examination. The coated specimen was then observed and photographed by scanning electron microscopy (Hitachi S-450, Japan).

#### *In vitro* release assays

25 mL 0.1 M phosphate buffer solution (pH 7.4), containing 0.5% sodium dodecyl sulphate (SDS) and 0.02% sodium azide, was added into a 30 mL tube, and then the drug-loaded microspheres (10 mg) were added into the tube and suspended thoroughly. The tube was placed in a 37±0.5°C water bath and shaken at 72 rpm speed horizontally [12]. At predetermined time intervals, 1 mL of medium was drawn out and replenished with the same volume of fresh medium. The withdraw medium was centrifuged at 5,000 rpm for 5 min, the supernatant was collected for determination, and the precipitate microspheres were put back to the testing tube. The dutasteride concentration was determined by an HPLC in triplicate.

#### Mass loss study

*In vitro* degradation study of drug-loaded microspheres was carried out in the same conditions used in the above release experiment. At the specific time point, the microspheres were collected by centrifugation (5,000 rpm, 5 min), washed three times with double-distilled water. After removing the upper clear solution, the microspheres were dried for 48 h in a vacuum oven to a constant weight. The samples were weighed and the weight reduction of the microspheres was evaluated by the formula shown below:
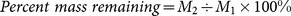



where, M_1_ is the initial mass of microspheres and M_2_ is the mass of the microspheres at predetermined time.

#### Gel permeation chromatography (GPC)

The molecular weights of the PLGA microspheres were determined by GPC (LC-20AT, Shmadzu, Japan) with a refractive index detector (RID-10A, Shmadzu, Japan). The TSK GEL GMHHR-N (7.8 mm×300 mm, Tosoh, Japan) analytical column was used. The mobile phase was tetrahydro-furan (THF) with a flow rate of 1 mL/min at 40°C. Microspheres (10 mg) were dissolved in 10 mL THF and filtered through a 0.22 µm filters. All glass equipment (vials and syringes) was used to minimize possible contamination from plastic materials. The polymer solution injection volume was 100 µL. Average molecular weights were calculated based on polystyrene standards (Mw from 480 to 950,000). The data collection and analysis were performed using LC Solution GPC software. Calibration was performed with every experiment. All the measurements were conducted in triplicate.

#### Biocompatibility

The biocompatibility of PLGA microspheres was assessed by MTT assay according to Wu et al. reported with minor modification [13]. Briefly, 0.1 g/mL blank PLGA microspheres in culture medium were incubated at 37°C for 24 h, then the extracts were filtered through a 0.22 µm membrane. L929 cell line (mouse connective tissue fibroblast cell) purchased from the Cell Resource Centre (IBMS, CAMS/PUMC) was maintained in Dulbecco's modified Eagle's medium (DMEM) supplemented with 10% fetal bovine serum, 2 mM glutamine, 100 µg/mL streptomycin and 100 IU/mL penicillin. The cells were maintained in a 37°C humidified incubator with a 5% CO_2_ atmosphere. The cells were harvested with 0.25% trypsin when cell monolayers reached more than 80% confluence. Then, cells were planted in 96-well plates at a density of 5000 cells per well with 200 µL DMEM per well. After 24 h incubation, the culture media were replaced with serial dilutions of the extracts. 24 and 96 h later, the cell viability was evaluated by MTT assay. In control wells, the culture media were replaced with fresh ones. Next, 20 µL of MTT (3-(4,5-dimethylfthiazol-2-yl)-2,5-diphenyl tetrazolium bromide) solution (5.0 mg/mL) was added to each well, and the plates were incubated for 4 h at 37°C. The media was removed and then 200 µL dimethyl sulfoxide was added to each well to dissolve the formazan crystals formed by the living cells. The absorbance at 570 nm of the solution in each well was recorded using a Microplate Reader (Model 680, BIO-RAD, USA). The relative cell viability (%) related to control wells was calculated by [Absorbance]_test_  =  [Absorbance]_control_ ×100.

#### Pharmacokinetics and pharmacodynamics studies. Animals and Treatments:

Male Sprague-Dawley rats (200±16 g) were purchased from Laboratory Animals Center of the 215th Hospital (China). All animals were handled according to the code of ethics in research, training and testing of drugs as laid down by the Animal Care and Use Ethics Committee of Academy of the 215th Hospital. All animals were pair-housed in polypropylene cages with free access to food and water. The vivarium was maintained on a 12 hour light: dark cycle (700∶1900) with a room temperature of 22±3°C and a relative humidity level of 50±5%.

#### *In vivo* release

Dutasteride-loaded microspheres used in *in vivo* release were sterilized by ^60^Co radiation, a common method used in sterilization of microspheres. Although exposure to ^60^Co radiation at an extent of 10 kGy will lead to <1% drug content lost, there was no effect on the release profiles of the microspheres [14].

The dutasteride *in vivo* release from the microspheres was studied in male Sprague-Dawley rats. 12 rats were randomly divided into two groups, the test group and a control group. In the test group, the back of each animal, where microsphere suspensions were injected subcutaneously using 21 gauge needles and administered at a dose equivalent to 8 mg dutasteride/kg in 1 mL dispersed solution (containing 0.5% CMC-Na and 0.05% Tween-80). In the control group, dutasteride solutions (dissolved in PEG400) were administered subcutaneously at a dose of 2 mg/kg [8].

All microsphere suspensions used were vortex-mixed before administration. After administration, all the centrifuge tubes were collected and freeze-dried to determine the residue drug and calculate the exact amount of drug injected.

Before and during the sampling time, the animals had free access to food and water. For control group, the sampling time was set as 0.03, 0.08, 0.25, 0.5, 0.75, 1, 2, 3, 4, 7, 12, 24 and 30 hours after administration; and for the test group, the time intervals was set as 0.17, 0.33, 0.5, 1, 2, 3, 5, 7, 14, 21 and 28 d after the injection.

At the designated time point, about 0.8 mL blood samples were collected from the retroorbital plexus of anesthetised rats, and transferred into heparinized tubes after drug administration. Plasma was separated by centrifugation (4°C) at 8,000 rpm for 10 min within 2 h. The plasma samples were frozen and stored at −20°C until analysis.

Dutasteride concentrations in the plasma samples were determined by an Agilent1200 liquid chromatography with tandem mass spectrometry (LC-MS/MS, Agilent Technologies, Santa Clara, CA, USA), as reported by Baek and Kim [15]. The lower limit of quantification of dutasteride was 0.5 ng/mL. The intra- and inter-batch precisions were less than 17% and their recoveries were greater than 80%.

The plasma concentration-time data were analyzed with noncompartmental model by WinNonlin 5.2 (Trial vision, Pharsight Corp., USA) to obtain the maximum plasma drug concentration (C_max_), terminal elimination rate constant (k_e_), terminal half-life (t_1/2_), area under the plasma concentration-time curve from time 0 to last point (AUC_0-t_) or infinity (AUC_0-∞_) and mean residence time up to last point (MRT_0-t_).

The correlation between the drug released (%) *in vitro* in PBS at 37°C and absorbed *in vivo* (F_a_) was examined. The F_a_ was determined using the Wagner-Nelson method by the following equation: F_a_ =  (C_t_/k_e_+AUC_0−t_)/AUC_0−∞_ [16]. The values of correlation coefficient (R^2^), slope and intercept were calculated, respectively.

#### Pharmacodynamics

A total of 40 male Sprague-Dawley rats were equally divided into four groups: Group 1 served as control (sham-operated group); Group 2 was model group; Group 3 animals were administered with dutasteride-loaded microspheres at an equal dose level of 0.756 mg/kg body weight; Group 4 served as a positive control group and treated with dutasteride solutions (a mixture of mono-di-glycerides of caprylic/capric acid and butylated hydroxytoluene) at a dose of 0.045 mg/kg, administered orally once daily for 28 consecutive days. The rats were weighed weekly during the experiments.

The BPH in rats were created by subcutaneous injection of testosterone (10 mg/kg) after castration [17], [18]. Briefly, the scrota of 10 rats were just cut open and then sewed up without cutting off the testicles as the sham group. Thirty other rats were castrated. The injection of edible oil was subcutaneously given to rats in the sham group, and testosterone was subcutaneously given to the castrated rats each day.

After a treatment period of 4 weeks and an overnight fasting, rats were anesthetized with chloral hydrate (300 mg/kg, i.p.) and blood samples were collected from the eye socket, centrifuged at 2000 rpm for 20 min to obtain serum for the determination of testosterone and DHT by RIA kits on a gamma ray counter. The prostates were collected from each rat and weighed. The wet weight index was calculated [19].

#### Statistical analysis

All data are shown as mean ± standard deviation (SD) unless particularly outlined. Student's t test or one-way analyses of variance (ANOVA) were performed in statistical evaluation. A P-value less than 0.05 was considered to be significant. All testing was done using the SPSS 13.0 (SPSS Inc., Somers, New York).

## Results and Discussion

### Formulation optimization

As dutasteride is a lipophilic compound and soluble in DCM with PLGA, the O/W solvent evaporation method was chosen to prepare PLGA microspheres in this study. In addition, emulsion solvent evaporation technique needs no special instrumentation [20] and only requires mild conditions, such as ambient temperature and constant stirring [21], which is widely used and preferable to other methods in preparing microspheres.

For the CCD, a total 20 trial formulations were proposed by Design-Expert software for three factors, PVA concentration (X_1_), PLGA content (X_2_) and theoretical content (X_3_), which were varied at five different levels. The effects of independent variables upon the drug encapsulation efficiency (%), mean particle size (µm) and cumulative drug release F_1d_ after 1 day (%) were investigated as optimization response parameters in this study. Overview of the experimental plan and observed responses were presented in Table 3.

**Table 3:** Experimental plan of central composite design with observed response values (n = 3).

	Factors employed			Observed responses		
Run	PVA concentration (X_1_, %)	PLGA content (X_2_, mg·mL^−1^)	Theoretical content (X_3_, %)	Encapsulation efficiency (Y_1_, %)	Mean diameter (Y_2_, µm)	F_1d_ (Y_3_, %)
1	4.50	99.43	5.00	70.3±2.1	48.9±5.4	14.5±6.3
2	5.37	259.00	6.80	65.9±1.5	29.4±3.1	22.2±11.2
3	4.50	199.50	8.02	60.6±1.2	41.8±3.9	26.0±15.4
4	5.37	140.00	6.80	58.6±2.9	34.7±2.5	21.6±10.7
5	3.04	199.50	5.00	79.2±2.4	74.7±7.0	13.8±7.6
6	4.50	299.60	5.00	81.6±3.3	54.3±4.2	14.3±8.4
7	5.96	199.50	5.00	72.6±1.7	23.1±3.8	16.5±9.1
8	4.50	199.50	5.00	74.3±2.8	44.5±3.2	15.2±8.7
9	3.63	259.00	6.80	78.4±2.1	62.1±5.5	20.4±9.2
10	4.50	199.50	5.00	75.3±1.6	44.6±3.9	14.9±6.98
11	4.50	199.50	1.99	69.2±1.8	42.6±4.7	8.3±3.1
12	5.37	259.00	3.21	78.6±2.5	31.2±4.1	11.1±6.2
13	5.37	140.00	3.21	76.1±1.7	33.8±4.9	10.8±4.9
14	3.63	140.00	3.21	86.2±1.4	46.3±5.3	10.1±5.0
15	4.50	199.50	5.00	76.4±1.2	44.3±5.2	13.5±7.4
16	4.50	199.50	5.00	73.9±1.3	46.1±5.6	14.3±7.1
17	4.50	199.50	5.00	74.9±1.9	45.8±5.8	14.2±6.5
18	3.63	259.00	3.21	89.2±1.8	69.7±6.4	9.4±7.7
19	3.63	140.00	6.80	62.4±1.4	45.1±4.8	21.0±9.4
20	4.50	199.50	5.00	73.5±1.3	42.0±5.3	14.7±8.9

The software proposes the following equations to describe model:
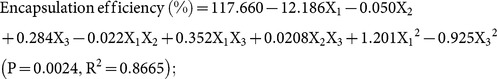


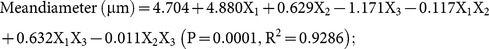


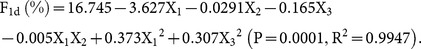



**Figure 1:**
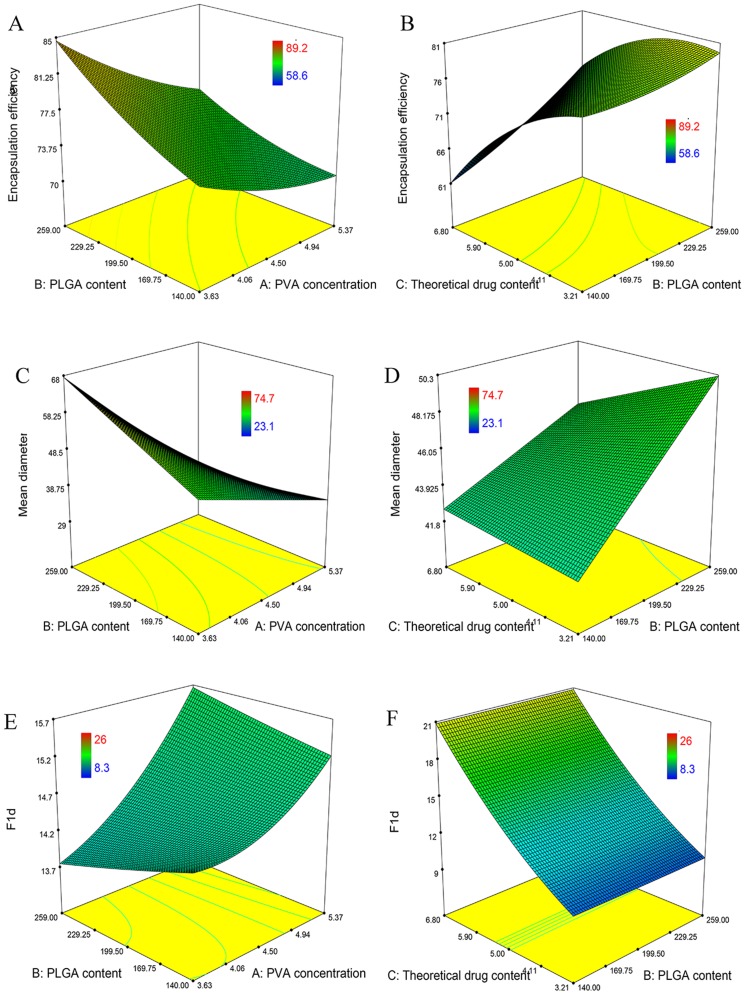
Three dimensional response surface graph of the relationship between independent and dependent variables. (A) Effect of PVA concentration & PLGA content on drug EE. (B) PLGA content and theoretical content on drug EE. (C) Effect of PVA concentration & PLGA content on particle size. (D) PLGA content and theoretical content on particle size. (E) Effect of PVA concentration & PLGA content on initial burst release. (F) PLGA content and theoretical content on initial burst release.

The three dimensional response surface graph is very useful in learning about the main and interaction effects of the independent variables. It should be notified that, the two ends of x axis in Fig. 1 respectively represented the level ±1 values (software default) of each corresponding factor in the Table 2, the values of level ±1.687 were ignored by the software automatically. As this did not affect the judgment of the overall trend here, we did not change the default setting of the software and re-produce the 3D plots. In addition, each x axis in Fig. 1 was quarterly divided into four parts by the software, and thus the values of the split-ticks on the x axis (except the two ends) were not the same as the data presented in Table 2 (level ±1 and 0).

The drug entrapment reflects the preparation efficiency, the higher the better. Fig. 1A and 1B described the relation between encapsulation efficiency (EE) and the formulation variables. As the PLGA content increased and PVA concentration decreased, the drug entrapment increased rapidly. When the PLGA content increased and theoretical content decreased, the drug entrapment increased and reached a maximum level.

In the solvent evaporation method, the drug EE reflects the partition of drug between disperse organic phase and continuous aqueous phase during the microspheres prepared process. As the PLGA content increased, the viscosity of organic phase would increase, so the drugs in the organic phase would have more difficulties in diffusing out and hence increased EE. The decrease of EE with the increase in PVA concentration may be explained by the resulting solubility enhancement of drug in the aqueous phase due to the presence of increasing quantities of PVA [22].

The particle size is an important property of microspheres, as it can influence the bio-pharmaceutical properties of the preparations [23]. Fig. 1C and 1D depicted the relationship between microsphere diameter and the formulation variables. The theoretical content almost had no effect on the particle size, while the PVA concentration would influence the diameter greatly. As the PVA concentration increased, the diameter decreased significantly. This was because the higher PVA concentration had greater reduction power on the interfacial tension, hence reducing the diameters of the initial droplets in the emulsions.

The initial burst release will influence the using safety of the deport form drugs, which needs special control in the microsphere preparations, and it is often attributed to the rapid dissolution of the drug that was deposited inside the pores or sticking to the surface of microspheres. Fig. 1E and 1F showed the relationship between initial burst release and the formulation variables. In this work, the percentage of initial burst release for all formulations ranged from 8.3% to 26.0%. The F_1d_ increased as the theoretical content or PVA concentration increased. A possible explanation was that the diameter decreased with the PVA concentration increased, smaller microspheres with higher surface areas would have higher surface drugs, thus resulting in a faster drug release. Moreover, as the theoretical content increased, there would be more drugs deposited in the pores of microspheres, so the initial burst release would increase.

Prediction profiler of the software was used to determine the optimum values of the factors for maximum EE, least particle size and minimum initial burst release. Finally, the first given optimum proposal (there were 27 proposals) was chosen, and the optimum values of PVA concentration (X_1_ = 5.37%), PLGA content (X_2_ = 259.00 mg·mL^−1^) and theoretical content (X_3_ = 3.21%) were obtained. These values predict drug entrapment efficiency (Y_1_ = 75.35%), particle size (Y_2_ = 29.97 µm) and initial burst release (Y_3_ = 11.22%).

### Prediction of the optimal formulation

These above predicted values of responses were validated by further conducting *in vitro* studies of dutasteride loaded microspheres prepared with previously optimized process parameters, and an average of 77.28% drug entrapment, 35.68 µm particle size and 11.83% initial burst release was obtained. This shows 102.6%, 119.1% and 105.4% validity of the predicted model for drug entrapment, particle size and initial release.

### Characteristics of optimized formulation

The optimal formulation identified via the CCD-RSM was characterized in terms of particle size, morphology, drug loading, *in vitro* release and *in vitro* degradation; the results are presented as follows.

### Morphology and particle size

**Figure 2:**
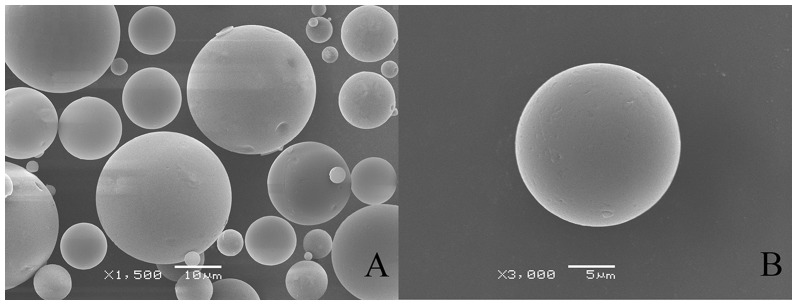
Scanning electron micrographs of dutasteride loaded PLGA microspheres.

The surface morphology of the microspheres was examined visually by SEM. As shown in Fig. 2, the prepared PLGA microspheres were spherical in shape and had a smooth surface with almost no pores or cavities, which may affect the release of encapsulated dutasteride. The microspheres are seen to be well formed and homogeneous, with no crystalline drugs and seldom fragments of polymer adhering.

**Figure 3:**
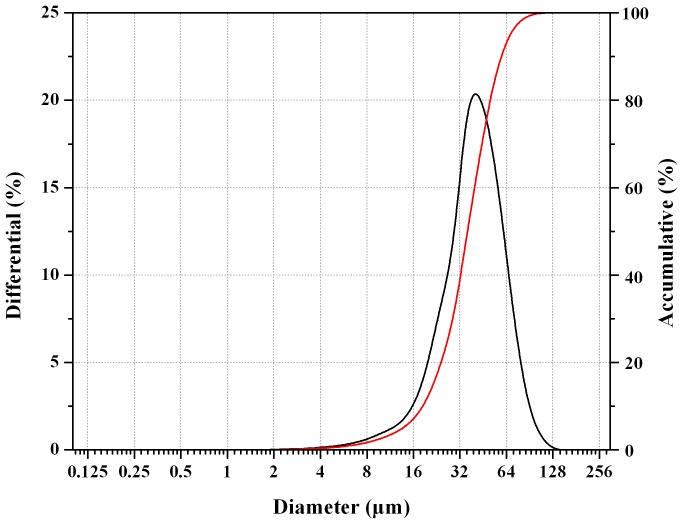
Particle size distribution of dutasteride loaded PLGA microspheres.

As shown in Fig. 3, the PLGA microsphere particles were mainly distributed around 40 µm, with the average value of 35.68±4.71 µm. Span was 1.10±0.09 (n = 3). These results demonstrated that the particle size of the prepared system was uniform and appropriate for subcutaneous injection.

### Injectability

Injectability of microspheres is an important criterion, so that microspheres can be administered through a needle of minimum inner diameter for a successful subcutaneous injection. Application of a maximum ejection force of 12 N over 10 s can be considered as a suitable development criterion. Suspensions of microspheres were injected through different needle diameters (24G, 21G and 18G). Experiment data (13.2±1.1, 9.3±0.7 and 4.9±0.4 N, n = 3, respectively) indicated neither partial nor complete blockage of the suspension flow. These results revealed that no injection difficulties were developed by using the prepared microspheres. Thus, they are suitable for subcutaneous injection through a 21G needle [11].

### Encapsulation efficiency and drug content

The encapsulation efficiency and drug content were 77.28±1.93 and 2.42±0.06% (n = 3), respectively. The un-encapsulated drugs were washed off in the preparation.

### *In vitro* drug release

**Figure 4:**
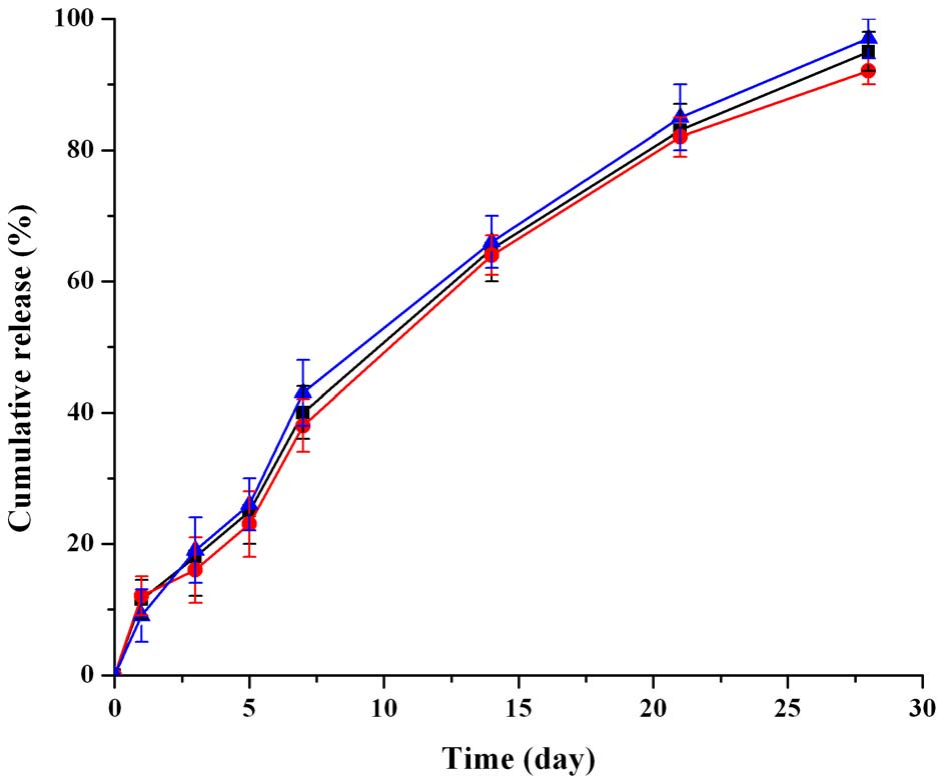
*In vitro* cumulative drug release from dutasteride loaded microspheres in 0.1 M PBS pH 7.4 at 37°C (n = 3).

The *in vitro* release of dutasteride from the microspheres depended greatly on the type of release medium. The effects of several factors, such as pH, buffer strength were investigated in preliminary studies and an appropriate *in vitro* release condition was found for this test. Fig. 4 showed the *in vitro* release profiles of three batches dutasteride loaded microspheres. The *in vitro* release behavior had a good reproducibility. After an initial burst phase of approximately 12% within the first 24 h, the release rate slowed down from day 2 to day 7; after that, a relatively fast release appeared and then slowed down gradually, over 90% drug was released by the day 28.

The *in vitro* release data were fitted using zero-order (F_t_ = 3.231t+11.59, R^2^ = 0.963), first-order (Ln(100 - Ft) = -0.101t+4.771, R^2^ = 0.973) and Higuchi (Q = 21.12t^1/2^ - 16.00, R^2^ = 0.985) models, respectively. As a result, the best fit was given by the Higuchi equation due to the highest correlation coefficient, which implied that the *in vitro* drug release was mainly controlled by diffusion mechanism [24].

### *In vitro* degradation of the microspheres

**Figure 5:**
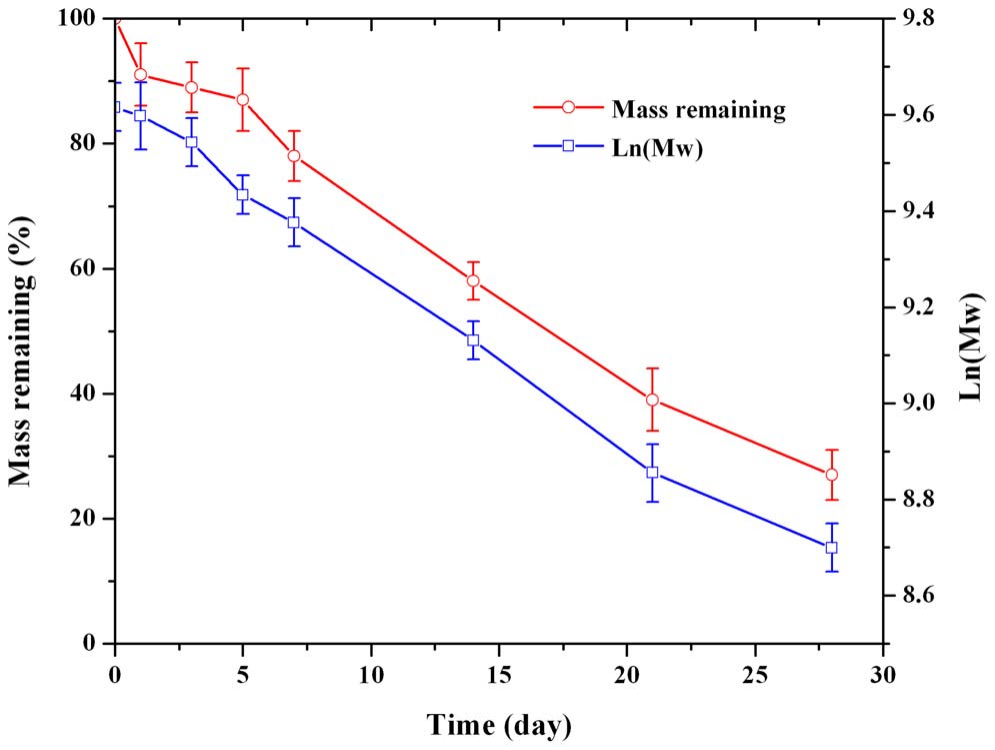
Mass remaining and molecular weight (Mw) change of dutasteride loaded microspheres in 0.1 M PBS pH 7.4 *in vitro* at 37°C (n = 3).

Fig. 5 displayed the mass loss profiles of microspheres during 28 days of degradation in PBS at 37°C. Initially, the weight remained relatively constant during the first 5 d. Then, the PLGA microspheres weight started to decrease rapidly, losing about 60% in 21 days. The molecular weight (Ln(Mw)) change of biodegradable microspheres with time showed a similar behavior. These results indicated that the mass loss process was occurred with the degeneration of polymers. In the lag release phase, significant amount of water soluble low molecular weight oligomers had been formed [25]. When these oligomers (with drug) were removed from the polymer matrix, mass losses began and another rapid drug release started.

### Biocompatibility

**Figure 6:**
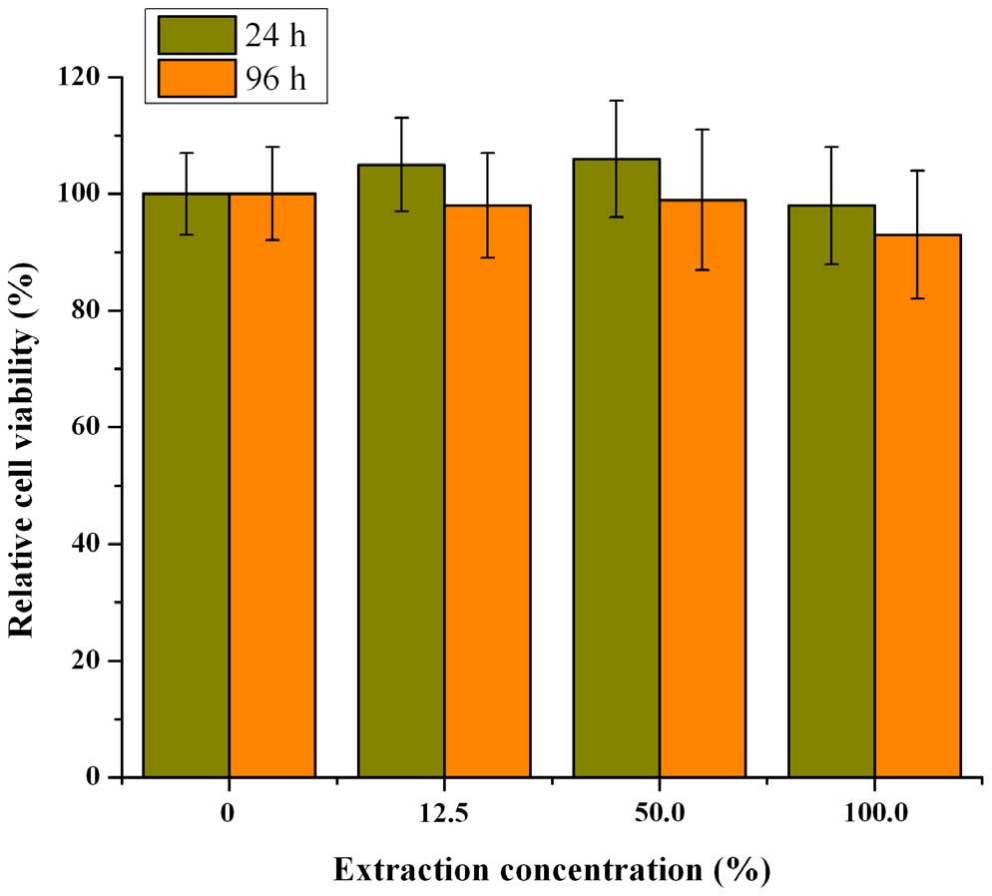
Relative cell viability (%) at various PLGA microspheres extraction concentrations (n = 6).

Relative cell viability from MTT assay was used to characterize the biocompatibility of the PLGA microspheres. As depicted in Fig. 6, no significant difference was found in L929 cell viability between the control and PLGA microsphere group at both 24 and 96 h. PLGA microspheres demonstrated low or no cytotoxicity, since their cell viabilities were all more than 80% even at the highest concentrations [26].

Biocompatibility is an important issue for applying biomaterials *in vivo*. Although the distinguishing biocompatibility of PLGA has been well accepted [27], it is still necessary to find out the toxic effect of the PLGA microspheres prepared. The MTT assay results suggested that the microspheres are biocompatible and can be used for controlled drug release.

### Pharmacokinetic

**Figure 7:**
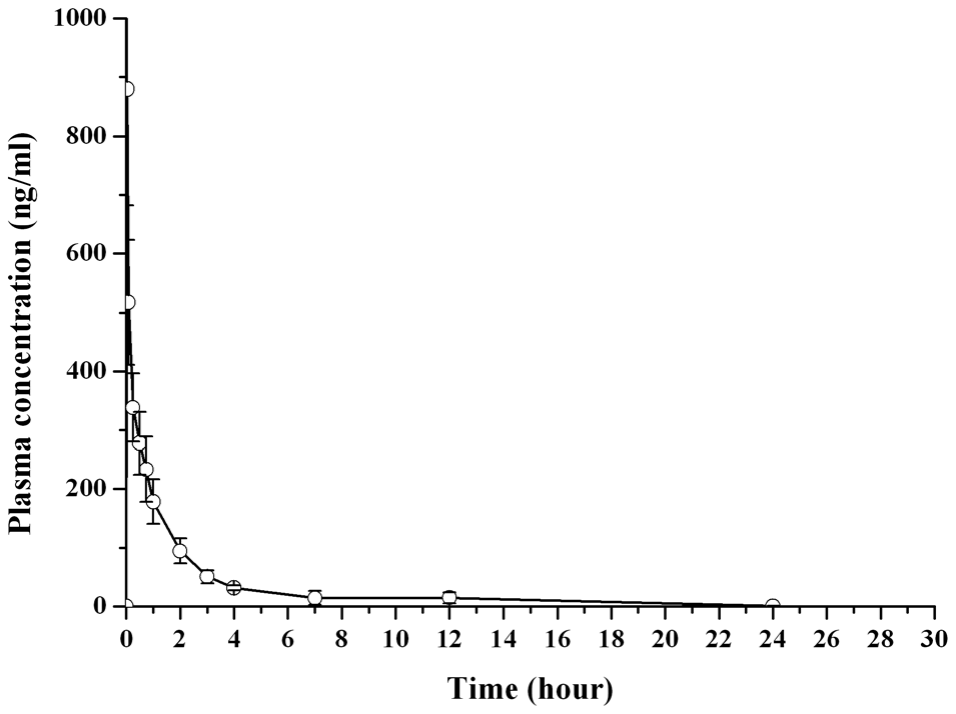
Drug plasma concentration versus time profile after subcutaneous injection of dutasteride solution to rats (n = 6).

**Figure 8:**
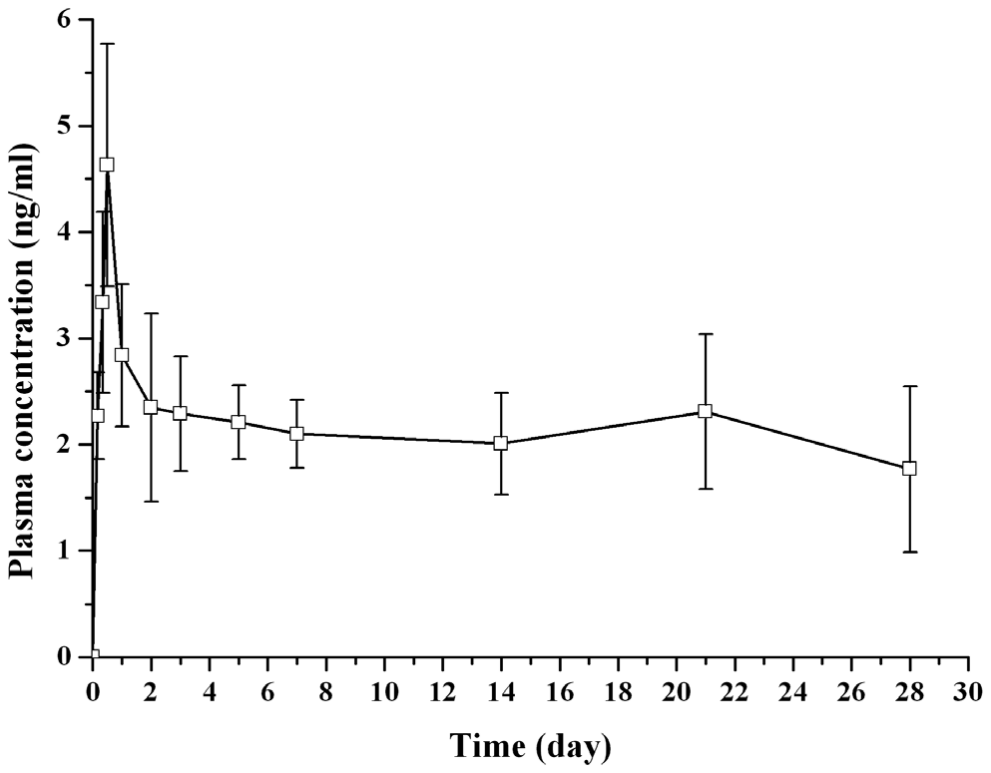
Drug plasma concentration versus time profile after subcutaneous injection of dutasteride microspheres to rats (n = 6).

Plasma concentration-time profiles of dutasteride after subcutaneous administration of dutasteride PEG400 solution and dutasteride encapsulated microspheres was shown in Fig. 7 and Fig. 8. As shown in Fig. 7, after subcutaneous injection of the drug solution, the peak plasma concentration of dutasteride (C_max_, 1321.20±296.73 ng/mL) reached within 3 min, and then decreased rapidly and remained about 10% of C_max_ after about 2.5 h, suggesting that the elimination of dutasteride was very fast. The peak value for dutasteride was 1321.2 ng/mL. By contrast, the *in vivo* profiles of drug-loaded microspheres were rather smooth (shown in Fig. 8). Drug plasma concentration was maintained at a relatively higher level (more than 2 ng/mL) in the initial 2 days after subcutaneous injection of dutasteride loaded microspheres. The concentration reached its maximum value (about 4.73 ng/mL) within the first 0.5 days. Drug concentration decreased markedly after 3 days, and then a relatively steady state concentration (about 2 ng/mL) for dutasteride was reached from day 7 to day 28.

The higher plasma drug level in the initial days could be attributed to the initial burst release of the microspheres. The microspheres in the current study release approximately 12% of the total dose in the 1^st^ day, this quantity is equivalent to 3.6 fold of the ideal daily release that was initially designed (i.e., 100% over 30 days, equivalent to 3.3% per day). It is worth to note that burst release may have dramatic consequences in case of low therapeutic index drugs [28]. However, according to the specifications of dutasteride soft gelatin capsule, 10 times of therapeutic dose (5 mg) for dutasteride was considered to be safe (60 subjects for 6 months) [29]. The burst release of the microspheres used in the current study is expected to be safe for clinical use. However, future studies will confirm this further.

The pharmacokinetic parameters are listed in Table 4. Obviously, much higher values of AUC, t_1/2_ and MRT, as well as much lower values in C_max_, were obtained for the drug-loaded PLGA microspheres, compared with those of drug solution. The AUC_(0-∞)_ of microspheres group was almost 4 times of the solutions group, this could be attributed to the dose difference between the two groups, the drug dose of microsphere group was 4 times of solution group. In order to meet the detection requirements of plasma drug concentration, the administration dose in this study was higher than that in the pharmacodynamics assay (calculated by body surface area conversion from human).

**Table 4:** Pharmacokinetic parameters after subcutaneous injection of dutasteride solutions and microspheres in rats (n = 6).

Parameter	Solutions (2 mg/kg)	Microspheres (8 mg/kg)
T_max_(h)	0.63±0.09	12.4±5.1
C_max_ (ng/mL)	1321.20±296.73	4.79±8.79
t_1/2_(h)	4.63±2.14	586.17±52.95
AUC_(0-t)_ (ng.h/mL)	776.19±103.74	1513.41±148.71
AUC_(0-∞)_ (ng.h/mL)	781.89±105.02	2969.32±395.84
MRT_(0-t)_ (h)	2.88±0.76	334.00±25.33

### The *in vitro* and *in vivo* correlation (IVIVC)

**Figure 9:**
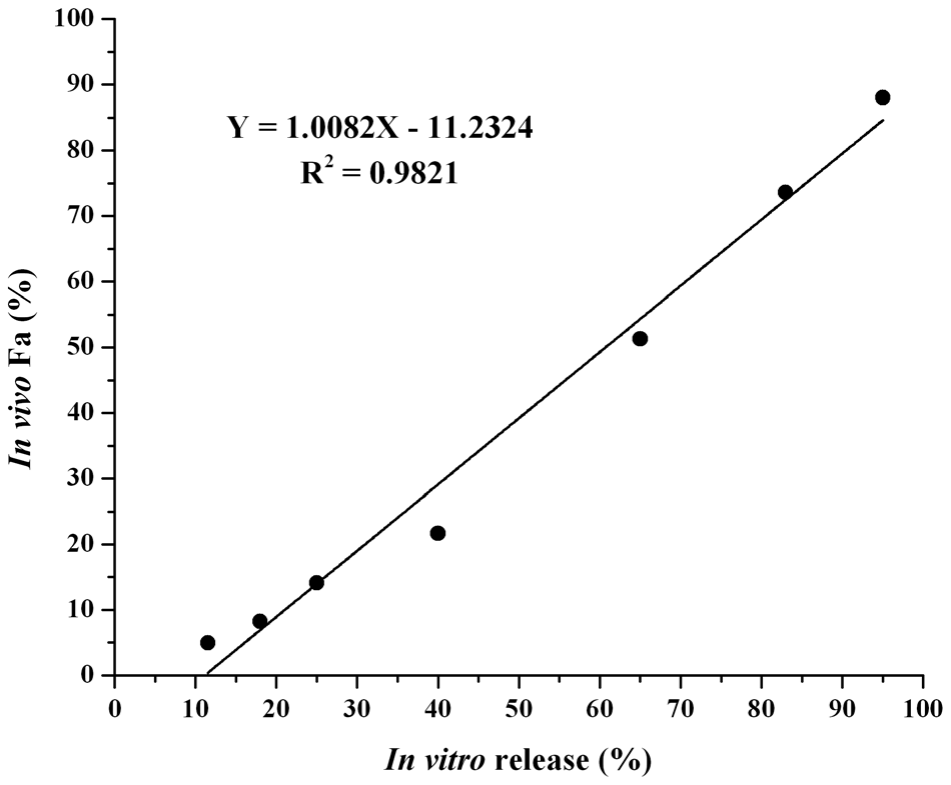
Linear regression plots of *in vivo* absorption versus *in vitro* cumulative release of dutasteride from PLGA microspheres.

The linear regression plot of drug released (%) vs. F_a_ for the dutasteride loaded microspheres was shown in Fig. 9, including the relative regression equation. A good linear regression correlation (R^2^ = 0.9821) was demonstrated between the percentage of drug released in PBS at 37°C and the percentage of drug absorbed from the microspheres in rats. This is a point-to-point relationship between *in vitro* dissolution rate and *in vivo* input rate of the drug from the dosage form, which belongs to the level A correlation [30]. Based on this result, it seems reasonable to predict the drug absorption *in vivo* by an *in vitro* release test.

IVIVC can be served as a surrogate for *in vivo* bioavailability and to support biowaivers, therefore it can be used in the development of new pharmaceuticals to reduce the number of human studies during the formulation development. IVIVC could also be employed to establish dissolution specifications for the quality control of products [31]. Therefore, it will be beneficial to develop an *in vitro* release test that is able to predict the *in vivo* absorption of dutasteride after subcutaneous injection. The data presented above suggest that the method using a shaken flask was acceptable in the IVIVC studies of subcutaneous microsphere formulation.

### Pharmacodynamics studies

Substantial evidence showed that androgens and DHT play a key role in the development of BPH. Dutasteride can suppress the blood DHT levels, and thus inhibit BPH [32]. Based on references reported [13], [33], at least 4 weeks were needed to demonstrate therapeutic effects on BPH rat models, so the pharmacodynamics index was measured 4 weeks later after dutasteride administration.

As shown in Table 5, the BHP model was successfully established. Compared with BPH model group, 14.5% (P<0.05) and 17.7% (P<0.01) decrease in gross prostate weight in rats administrated with microspheres and solutions for 4 weeks was observed, respectively. There were no significant differences between microsphere group and solution group.

**Table 5:** The effects of different treatments on the prostate weight of BPH rats (n = 10).

			Prostate weight (g/g×100)		
Group	Weight (g)	Lateral lobe (g)	Ventral lobe	Retral lobe	Gross weight
Sham group	395.2±30.5	0.10±0.03	0.14±0.03	0.07±0.02	0.31±0.06
Model group	334.8±17.1	0.22±0.04**	0.29±0.03**	0.12±0.02**	0.62±0.04**
Microsphere group	341.5±13.2	0.18±0.06	0.23±0.05△	0.11±0.07	0.51±0.07△
Solution group	340.1±13.9	0.17±0.05	0.22±0.04△	0.11±0.05	0.53±0.08△

**P<0.01 vs. sham group.

△ P<0.05 vs. model group.

After 4 weeks, the administration of dutasteride solutions and microspheres caused a decrease in serum DHT levels from 0.365±0.047 ng/mL in the BPH group to 0.164±0.019 and 0.158±0.021 ng/mL (P<0.05), respectively (Table 6). There was no statistics difference between microsphere group and solution group on DHT and testosterone levels in serum. Moreover, there were no statistically significant changes in the testosterone concentration among the four groups.

**Table 6:** The effects of different treatments on DHT and testosterone levels in serum (n = 10).

Group	Testosterone (ng/mL)	DHT (ng/mL)
Sham group	1. 913±0.614	0.087±0.016
Model group	9.878±0.911*	0.365±0.047*
Microsphere group	9.376±1.413	0.158±0.021△
Solution group	9.635±1.408	0.164±0.019△

*P<0.05 vs. sham group.

△ P<0.05 vs. model group.

These results suggest that the prepared extended-release microspheres demonstrated the same inhibitory effect on BPH, compared to orally (once per day for 28 consecutive days) administered dutasteride.

## Conclusions

Dutasteride-loaded PLGA microspheres were successfully prepared by an O/W emulsion solvent evaporation method. RSM was used to optimize the formulation of microspheres, which predicted the values of independent/dependent variables within the experimental range and had a good agreement between the predicted and experimental values. The optimized microspheres were able to provide a long-term release of dutasteride. It was proved that the preparation process was reproducible, and the PLGA particles obtained were spherical in morphology and appropriate in size. A steady drug release from the microspheres was observed *in vitro*, which can sustain for about 28 days and was well fitted with Higuchi model. Similarly, after the subcutaneous injection of drug loaded microspheres to rats, about 1 month long plasma drug concentration versus time profile was observed in the *in vivo* study. A good IVIVC was found and established. In pharmacodynamics studies, the microsphere formulation showed similar therapeutic efficacy on BPH inhibition, when compared to the oral dutasteride solution that was administered once daily for 28 consecutive days. Based on these results, it can be concluded that the prepared PLGA microspheres are quiet hopeful to continuously delivery dutasteride for 1 month in the future clinical application.
